# An Early Warning System for Flood Detection Using Critical Slowing Down

**DOI:** 10.3390/ijerph17176131

**Published:** 2020-08-24

**Authors:** Syed Mohamad Sadiq Syed Musa, Mohd Salmi Md Noorani, Fatimah Abdul Razak, Munira Ismail, Mohd Almie Alias, Saiful Izzuan Hussain

**Affiliations:** Department of Mathematical Sciences, Faculty of Science and Technology, Universiti Kebangsaan Malaysia, 43600 Bangi, Selangor, Malaysia; msn@ukm.edu.my (M.S.M.N.); fatima84@ukm.edu.my (F.A.R.); munira@ukm.edu.my (M.I.); mohdalmie@ukm.edu.my (M.A.A.); sih@ukm.edu.my (S.I.H.)

**Keywords:** critical slowing down, water level, quantile, flood early warning system

## Abstract

The theory of critical slowing down (CSD) suggests an increasing pattern in the time series of CSD indicators near catastrophic events. This theory has been successfully used as a generic indicator of early warning signals in various fields, including climate research. In this paper, we present an application of CSD on water level data with the aim of producing an early warning signal for floods. To achieve this, we inspect the trend of CSD indicators using quantile estimation instead of using the standard method of Kendall’s tau rank correlation, which we found is inconsistent for our data set. For our flood early warning system (FLEWS), quantile estimation is used to provide thresholds to extract the dates associated with significant increases on the time series of the CSD indicators. We apply CSD theory on water level data of Kelantan River and found that it is a reliable technique to produce a FLEWS as it demonstrates an increasing pattern near the flood events. We then apply quantile estimation on the time series of CSD indicators and we manage to establish an early warning signal for ten of the twelve flood events. The other two events are detected on the first day of the flood.

## 1. Introduction

Various fields of scientific works are now suggesting the existence of early generic warning signals as an indicator when systems are approaching their critical tipping points [1]. The climate is one of the complex dynamical systems having tipping points. At those tipping points, the climate may experience shifts to different dynamical regimes [2]. The suggested generic indicators, when the climate gets close to its critical tipping point, are related to the theory of critical slowing down (CSD) [3]. The theory of CSD explains that as a tipping point is approached, an increasing pattern in the time series of the CSD indicators is expected to occur. Two possible CSD indicators of early warning signals are increased in variance [4] and spectral density [5].

These generic early warning signals had captured the essence of shifts in tipping points in several natural systems ranging from ecosystems [6,7] to climate [1,2,8] and financial systems [9,10,11]. All of the above researches show that there is an increasing pattern in the time series of the indicators near the tipping points. Previous studies [8,11] investigated the increasing pattern using Kendall’s tau rank correlation, but their results showed inconsistency as the strength of the correlation varied among events.

In hydrology, an early warning system (EWS) that will alert when the next flood is going to happen is very crucial. As flood is a destructive natural disaster, it is responsible for a high number of deaths and loss of property. Even though floods are unavoidable, a flood early warning system (FLEWS) is important to anticipate a flood and its impacts. Hence, FLEWS should be developed using reliable methods for better prediction and flood preparation. Since flooding is defined in general as an overflowing of water onto land that is generally dry, an understanding of observational and historical water level data is essential as they provide climatic indicators for flooding.

Most research on FLEWS or flood forecasting is done by constructing models of hydrological processes. Previous studies on FLEWS focused on rainfall–runoff models; see [12] for worldwide EWS review and reference [13] for Malaysia EWS review. The current research trend is to integrate new techniques into rainfall–runoff models. For example, artificial neural network modeling and entropy theory have been used to gain enhanced rainfall–runoff models [14,15,16,17]. For some other studies, physical models have been developed for flood forecasting [18,19].

In this paper, we consider the CSD approach to construct a FLEWS using water level data. As a case study, we analyze the time series data of the daily water level at the Guillemard Bridge station, Kelantan River, Malaysia, from 1 January 2000 to 13 October 2010. Based on the time series of water level data, we calculate the CSD indicators and inspect the increasing pattern. Since the result obtained through the standard analysis method of Kendall’s tau rank correlation is unfavorable for our data set, we propose a new analysis method using quantile estimation. Quantile estimation is a method used in extreme value theory that has been used by hydrologists to study rare events and extreme values [20,21]. Through getting an optimum quantile, we will gain thresholds that will justify the significant increase in the CSD indicators and date extraction for the EWS.

In Section 2, we provide a concise and informal review of CSD as an early warning signal with the construction of EWS through Kendall’s tau rank correlation and quantile estimation. In Section 3, we introduce our water level data. Section 4 presents our analysis and results and Section 5 concludes the paper.

## 2. Materials and Methods

This section provides an introduction for EWS using the theory of CSD and method of analysis for the detection of increasing patterns in the time series of CSD indicators, Kendall’s tau rank correlation versus quantile estimation.

### 2.1. Early Warning System Using Critical Slowing Down

EWS is a tool consisting of a series of mechanisms and procedures for detecting hazards, monitoring indicators, warning communications, and alarms. A study has been conducted to provide efficient EWS [12,13]. However, developing an EWS based on real data is challenging and may lead to false-positive results as well as false-negative results. False-negatives are situations in which a sudden shift occurred, but no early warning signal could be detected before the shift. While a false-positive occurs if a supposed early warning signal is not the result of an approaching hazard, which is also called a false alarm. Hence, the development and implementation of EWS need to enhance its performance.

Various scientific works suggested CSD as an indicator of EWS. This type of slowing down is measured as increases in variance [4] and spectral density [5] can be shown to be a typical characteristic of a system approaching its tipping points. In the earth system, an irregular shift in ocean circulation and climate may occur. The proposed explanations for this abrupt climate change usually invoke the existence of thresholds in external conditions, where the climate system reached its tipping point. In a recent analysis [8], a significant increase in each of the eight ancient abrupt climate changes shows that they were all preceded by the characteristic of CSD in the fluctuation starting well before the actual shift.

Note that hydrologists have a long history of research using indicators such as autocorrelation, variance, and power spectrum to study hydrological data [22,23] even though not for critical slowing down or early warning system purposes. We also found one paper [24] that uses the theory of critical slowing down in the hydrological field to quantify the long-term hydrological shifts based on river discharge, however, the authors just used one indicator which was autocorrelation for their analysis. Further, throughout this study, the term of an early warning signal is meant by any signal that is determined before the day of the flood event. By this definition, signals detected on the day of the event are not considered an early warning signal but labeled as detection on the first day. Of course, signals detected after the events are considered a false alarm.

### 2.2. Kendall Tau Versus Quantile Estimation

The standard way to investigate whether there is an early warning signal on the CSD indicators is to test the trend over time for significance using Kendall’s tau rank correlation [8,10,11]. In this study, the usage of Kendall’s tau rank correlation is a replication of the work by Guttal et al. [10]. Kendall’s tau rank correlation measures the correlation of the time series of CSD indicators with an increasing sequence. The value of Kendall’s tau ranges between [−1, 1] and will determine whether the CSD indicators show an increasing or decreasing pattern. A positive value of Kendall’s tau indicates an increasing pattern of the CSD indicators, while a negative value indicates a decreasing pattern. A high value of Kendall’s tau suggests a strong trend. In the presence of CSD, one expects to find a significant increase trend as indicated by a significantly positive value of Kendall’s tau, 0.9 and above. However, some studies [10,11] show that the result of Kendall’s tau obtained is inconsistent. Their result shows that all the trends were significant (positive value) as measured by Kendall’s tau, but the strength of the correlation varied among events.

Therefore, here we propose a different approach for the analysis of an early warning signal from the CSD indicators to tackle the issue of inconsistent detection through Kendall’s tau rank correlation method. After observing the increasing pattern on the time series of the indicators, we attempt to find the threshold that justifies the trend. As the increasing patterns of the indicators are happening because of the extreme values in the time series, we use quantile estimation to come out with the threshold value that will justify the patterns. This threshold value will tell us that the data that exceeded the value are considered extreme and responsible for the increasing pattern. Quantile estimation is a method in extreme value theory to study extreme values and rare events. Hydrologists also have used this method to study anomalies in hydrological data [20,21].

## 3. Data

The Malaysian climate is governed by two main regimes: the southwest and northeast monsoons [25]. The southwest monsoon occurs between May and August and is responsible for the dry period for the whole country. The northeast monsoon usually starts in November and ends in February, which is responsible for the wet period (heavy rains) on the east coast of Peninsular Malaysia and frequently causes monsoon flooding.

Kelantan is one of the states that is located on the east coast of Peninsular Malaysia and is often affected by monsoon flooding. Kelantan River is located in the northeast of Peninsular Malaysia between the latitudes 4°40′ and 6°12′ north, and longitudes 101°20′ and 102°20′ east. It is the longest river in Kelantan at 248 km and drains an area of 13,100 km^2^. The total area of Kelantan is 15,022 km^2^. Furthermore, approximately 68.5% of the population lives in the Kelantan River basin. The Kelantan River originates from the Tahan mountain ranges and flows into the South China Sea moving northwards. The Kelantan River has two tributary rivers: the Galas River, and the Lebir River. The Galas River has two main tributaries (the Nenggiri River and the Pergau River), while the Lebir River has one major tributary (the Relai River).

Annual precipitation over the Kelantan River basin ranges from 0 mm in the dry season from March to May to 1750 mm in the rainy or monsoon season from November to January. For this region, the estimated runoff is 500 m^3^ s^−1^. The Kelantan catchment has various types of soil but is dominated by sedentary soils on hills and mountains, while on riverine floodplains and low riverine terraces, alluvial soil appears. Agriculture (paddy, rubber, and oil palm) for midstream and downstream and upstream forests (i.e., near Gua Musang) are the main land uses of this region.

The Kelantan River frequently overflows when the northeast monsoon reaches Kelantan with heavy rains, triggering an almost annual recurrence of monsoon flooding [26]. Furthermore, rapid land use changes were reported from the 1970s to the 2000s, especially concerning deforestation (due to logging activities) and conversion to agricultural land (rubber and oil palm) [27,28]. A recent study [29] reported that land use and climate change (i.e., precipitation) have a substantial effect on hydrological processes in the Kelantan monsoon catchment, but that precipitation changes are the main driver for the downstream catchment, which is more vulnerable to flooding, with related possible socio-economic impacts.

The River Kelantan has become increasingly susceptible to flood disasters, and this is potentially due to meteorological factors such as climate change, rapid changes in land use, and weaknesses in development planning and monitoring. The River Kelantan is important because it is subject to the most severe monsoon flooding in Malaysia. In Malaysia, the first recorded major flood event occurred in 1886 and had caused extensive damage in Kelantan, Malaysia [25]. Based on the report by the Department of Drainage and Irrigation, DID [30], on the flood events at Kelantan, starting with the year 2000, the first severe flood that hit Kelantan was reported in December 2001 due to the unusual tropical cyclone Vamei. Afterward, in the years 2007 and 2009, heavy rainfall again had triggered major floods in Kelantan. To date, the worst flood reported in Kelantan was at the end of 2014, commonly known as the Kelantan Big Yellow Flood 2014 [31].

**Figure 1:**
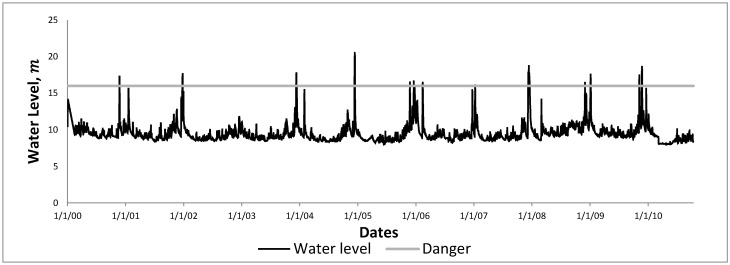
Time series plot of daily water level data of Kelantan River at the Guillemard Bridge station from 1 January 2000 until 13 October 2010.

Therefore, in this research, our analysis focuses on water levels in the Kelantan River. The daily water level data of Kelantan River recorded at the Guillemard Bridge station (measured in meters, m) were obtained from the DID. The DID has assigned a water level of 16 m as the danger of the Kelantan River at the Guillemard Bridge station as an indicator of a flood. Figure 1 shows a time series plot of the daily water level data of the Kelantan River obtained from 1 January 2000 until 13 October 2010. Some important statistical parameters of the time series are shown in Table 1. Table 2 lists the dates in which the water level data exceeded the danger level (16 m); these dates will be used as benchmark dates for flood events.

## 4. Results and Discussion

This section is divided into two parts. The first part will discuss the results of CSD indicators from the water level data of Kelantan River. The purpose of this part is to visualize the trend of the indicators. In the second part, we will analyze the trend of the CSD indicators to produce a FLEWS using Kendall’s tau rank correlation (standard way) and quantile estimation (proposed method).

### 4.1. Critical Slowing Down for Early Warning Signal

**Figure 2:**
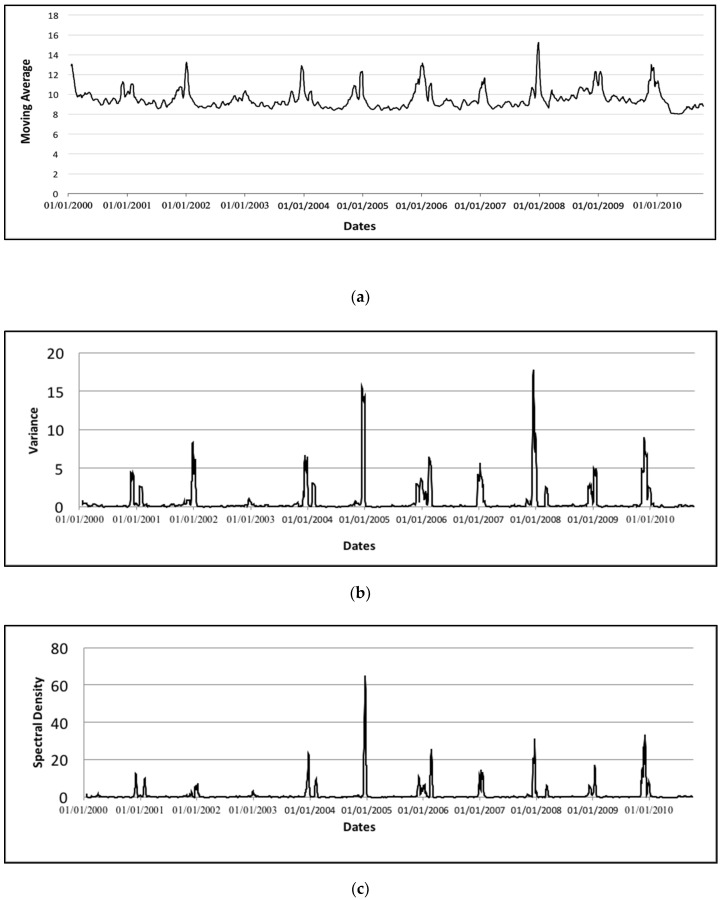
Time series of moving average and critical slowing down (CSD) indicators: (**a**) the time series of twenty days’ moving average of the water level data of Kelantan River; (**b**) the variance of the water level data of Kelantan River; (**c**) the average spectral density at low frequencies of the water level data of Kelantan River.

To produce an EWS, here we use the water level data in hand as a basis and compute the CSD indicators to come out with early warning signals for the flood. We employ twenty days’ size of the sliding window with a time step of one day to calculate the moving average, variance, and average spectral density at low frequencies for each window of the water level data. This twenty days’ size of the sliding window used to calculate the CSD indicators is suitable as it manages and provides us with the increasing pattern in the time series of indicators. Even if we change the window size to ten days’ size, we will still manage to obtain the increasing pattern on the CSD indicator time series. Therefore, this size of the sliding window is not really sensitive as long as we can get the increasing pattern on the CSD indicators so we can proceed with the EWS. Figure 2 shows the time series of the moving average and the obtained time series of the indicators, variance, and the average spectral density at low frequencies for each window of the water level data. This figure visualized the trend of the moving average and the CSD indicators. Figure 2 indicates that at the end of each year or the beginning of the following year (wet period from November to February), there is an increasing pattern on the moving average and both indicators. It proves that the CSD indicators produce a significant warning signal for the flood events of each year.

**Figure 3:**
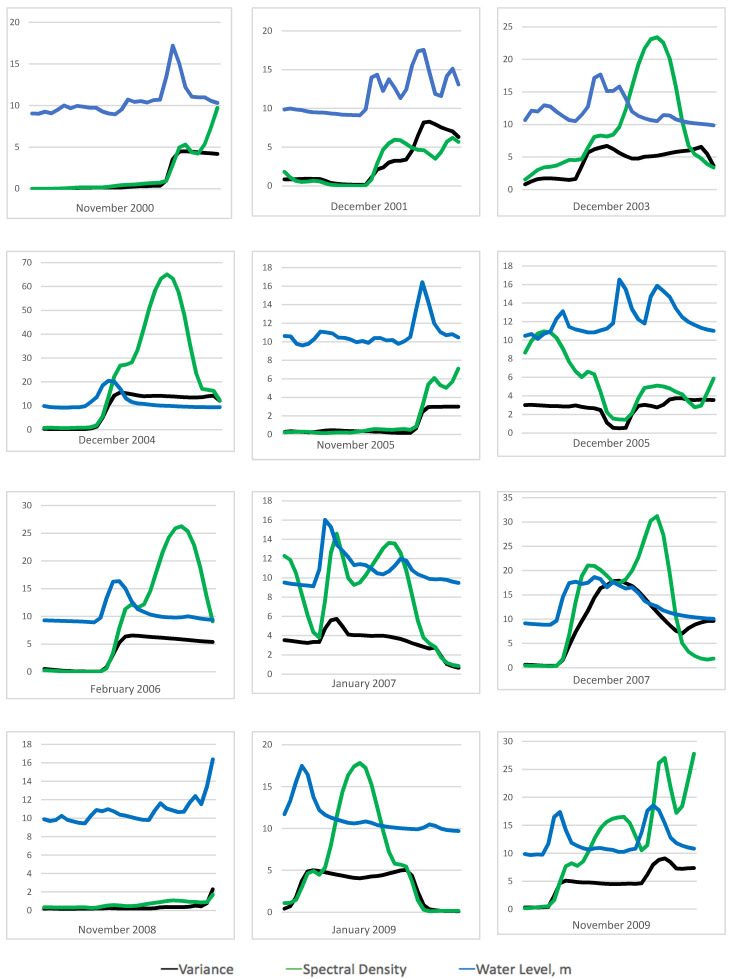
Trend of the time series of water level and the time series of the critical slowing down indicators, variance, and the average spectral density at low frequencies for all twelve flood events at Kelantan River from 1 January 2000 until 13 October 2010.

Figure 3 shows the close up of the CSD indicators for all twelve flood events at Kelantan River from 1 January 2000 until 13 October 2010 (Table 2) and their respective water level time series. It shows that both indicators are increasing with fluctuation patterns near flood events of every year. Simultaneously, the increasing pattern shows that both indicators agree with the water level data. The increasing pattern as observed in the time series of the CSD indicators can be used to produce a FLEWS.

### 4.2. Kendall’s Tau Versus Quantile Estimation for Early Warning System

Here, we examine the increasing pattern of the CSD indicators from the water level data using Kendall’s tau rank correlation and quantile estimation for a more effective and efficient EWS. Even though we can see in Figure 3 that near the flood events, both indicators are increasing simultaneously, after we calculate Kendall’s tau value, the strength of the correlation is varied for both indicators. For the calculation of the Kendall’s tau rank correlation, here we applied ten days’ window size to calculate the Kendall’s tau rank correlation. This is a sensitive window size because it will affect our EWS if chosen wrongly as it determines the number of early warning signals and false alarms gained. We already considered smaller and bigger window sizes to calculate this Kendall’s tau value, but the result obtained shows inconsistency as a smaller window size will give us more windows with a strong correlation, resulting in more false alarms. A bigger window size will affect our detection of the flood events. For example, flood frequently happens in Malaysia. The big window size will detect two flood events as one, not separately.

However, based on the results obtained by Kendall’s tau rank correlation analysis, the dates with a significantly strong correlation are different for both indicators. Table 3 shows the result of the EWS from the water level data determined by Kendall’s tau exceeding a threshold value of 0.9 for each CSD indicators’ variance and average spectral density at low frequencies. Note that for this label for the result through Kendall’s tau rank correlation, we label the outcome following Guttal et al. [10] based on the strength of the correlation on the time series of CSD indicators. The nearest warning signal will be assigned to respective flood events. If there are warning signals with no corresponding flood events, then they are considered false alarms.

Based on the results from Table 3, the variance succeeded in performing an early warning signal for seven of twelve flood events. However, some signals cannot be detected during actual flood events. Surprisingly, some of them are falsely detected during the dry period of May until August: flood events December 2004 and January 2007. In other cases, there are no signals and some with a late signal took between two to six months to be detected: December 2005, February 2006, December 2007, January 2009, and November 2009. The result of the EWS based on the CSD indicators of the average spectral density at low frequencies succeeded in performing five of twelve early warning signals for flood events. Nevertheless, the issue stays the same as some signals happen too early during the dry period, some are late, and some are not detected: flood events December 2001, December 2004, November 2005, December 2005, February 2006, November 2008, January 2009, and December 2009.

All the errors show that flood events cannot be detected on the exact dates even though it can be clearly seen from Figure 3 that there is an increasing pattern in the CSD indicators during the flood events. These results suggest that the measurement of Kendall’s tau rank correlation is inconsistent with detecting the increasing pattern in the CSD indicators for our data set. Therefore, we proposed a new analysis method to verify the increasing pattern of the CSD indicators to come out with a better EWS. We use a method in extreme value theory known as quantile estimation to come out with a threshold that justifies the significant increasing pattern. This quantile will provide us with threshold values of the CSD indicators so that when we peak over the thresholds, we will get dates consisting of the extreme values that are responsible for the increasing pattern. As we can see from Figure 2, the peaks for both indicators are arriving almost at the same time and this indicates the same flood events in the water level data. Therefore, in this study, we use the same number of the quantile for both indicators (to prevent more signals detected for different indicators), and the dates of the signal for flood events extracted should exceed the thresholds for both indicators at the same time (to prevent false alarms that are created through the signal from just from one of the indicators). Hence, the result of an early warning signal obtained should interpret that the increasing pattern occurs on both indicators at the same time.

To obtain the suitable or optimum quantile to extract the dates with extreme values, here we list down all possible values of the quantile and look for the best result of the EWS. Table 4 lists down the results of the EWS from the water level data for different values of the quantile with their respective weights. We can see that as the quantile value increases, the number of early warning signals obtained also increases together with the number of false alarms, while the number of late signals decreases. We explored many numbers for this quantile value. In addition, it seems that the range 10% to 20% is the suitable value for the optimum quantile. For an explanation, Table 4 shows that at 10%, the quantile number of the EWS obtained is two and it already shows a less efficient EWS. So, there is no need to lower the number of the quantile value as it will only come out with a less effective EWS. At the quantile 20%, the number of false alarms is nine, which is more than half of the total number of flood events (twelve flood events). This number of false alarms also shows a less efficient EWS. If we continue to increase the quantile value, it will only create an EWS with more false alarms.

At the quantile 15%, the number of early warning signals obtained starts to be stationary for quite some time together with the number of late signals, while the number of false alarms continues to rise. This indicates that the quantile 15% is the optimum quantile to gain the best result for the EWS. This result is further verified by assigning weight to each outcome of the EWS (0.5 for an early warning signal, 0.3 for a late signal, and −0.2 for a false alarm) and calculating the total weight of the outcome for each quantile. We found the highest weight of 4.4 is at the optimum quantile 15%, and this proves the result obtained. Regarding the weight applied, the biggest weight of 0.5 is assigned to the outcome of early warning signals as it shows the effectiveness of the EWS. For a late signal (or detection), we assigned the weight 0.3 as this outcome is expected for some flood events. Lastly, we take the weight −0.2 for false alarms, as these false alarms have a negative impact in terms of the efficiency of the EWS.

Table 5 shows the result of the FLEWS at the optimum quantile of 15% with threshold values of 0.4742 for the time series of the variance and 0.7909 for the time series of the average spectral density at low frequencies. This threshold value gives us eighteen signals. Twelve of the signals show that this framework succeeds in detecting all twelve of the events: ten events with an early warning signal, while two other events are detected on the first day of the flood. The other six signals correspond to false alarms, with rates of 33.33%. Note that for this quantile estimation approach, we label the outcome of the signal depending on the interception of the signal with the dates of the flood events. Based on the date of the signal, we will decide whether there is an early signal or late signal (or detection). If there are no corresponding flood events during the warning signal, then it is considered a false alarm.

In detail, for the early warning signals, a different number of days with a range between one and eighteen days was established in this study for the floods’ early warning. This range of early warning is more significant for flood compared to early warning that takes months, produced via Kendall’s tau rank correlation in Table 3. One-day early warning signals are obtained for flood events November 2000, December 2004, November 2005, December 2007, and November 2008, while early warning signals for the rest of the flood events are between three and eighteen days (flood event January 2007). There is no event that occurs without a signal, and the lowest is the detection on the first day of the flood from flood events February 2006 and November 2009. This FLEWS also created six false alarms, in which during these false alarms, signals were obtained in the time series of CSD indicators while there were no flood events during the time period (i.e., signal on 19/01/2001 and 16/11/2001).

In conclusion, after we change the method of analysis from the standard method of Kendall’s tau rank correlation to quantile estimation, we found that the FLEWS created becomes more significant. This is because the early warning signals were obtained just a few days before the flood events or on the same date, not up to six or nine months, and for some cases late warnings were observed through Kendall’s tau rank correlation. Further, all the early warning signals are shown on both CSD indicators, which shows consistency compared to the FLEWS through Kendall’s tau rank correlation where some of the signals are just shown on one of the indicators. Other than that, using the proposed method, all flood events are detected compared to the standard method that was not able to detect three flood events (December 2005, December 2007, January 2009) (see Table 3). However, the number of false alarms created is still the same—six false alarms.

## 5. Conclusions

In this work, we successfully applied the theory of CSD to the hydrological field to analyze water level data for the early warning of flood disasters at Kelantan River. The increasing pattern in the time series indicators of CSD (variance and average spectral density at low frequencies) is a symptom of the warning signal. We also proposed the method of quantile estimation to analyze the increasing pattern to find the threshold that justifies the significant rising trend of the CSD indicators since the standard way of analysis using Kendall’s tau rank correlation is not favorable for our data set.

In a case study on water level data from Kelantan River, we found that the time series of water level data exhibit CSD by demonstrating the increasing pattern near the flood events. From the quantile estimation, the optimum results are at the quantile 15% with threshold values 0.4742 for the time series of variance and 0.7909 for the time series of average spectral density at low frequencies. These thresholds succeeded in detecting all twelve flood events and producing ten early warning signals for the floods events and the other two flood events were detected on the first day of the flood. These thresholds also created six false alarms.

In conclusion, this study suggests that the theory of CSD is a reliable indicator to perform a FLEWS at Kelantan River. The method for finding the threshold using quantile estimation also shows consistency to detect all the increasing patterns on the CSD indicators near all the flood events and produce an early warning signal for the majority of the flood events. The drawback of this study, which can be strengthened for future studies, is that the analysis could be achieved by first splitting the data into calibration and validation sets to measure how the method’s performance in future floods will be. Besides, more research should be conducted to see the full performance of the proposed methods under different climate conditions or watershed sizes.

## Figures and Tables

**Table 1:** Statistics for the time series of daily water level data of Kelantan River at the Guillemard Bridge station from 1 January 2000 until 13 October 2010.

Statistics	Daily
Number of data	3939
Average	9.52
Max	20.44
Min	8
Standard deviation	1.26
Skew	3.29
Kurtosis	15.64

**Table 2:** List of dates of the water level data that exceeded the danger water level (16 m) of Kelantan River at the Guillemard Bridge station from 1 January 2000 until 13 October 2010.

No.	Date of Flood Events	No.	Date of Flood Events
1.	23/11/2000	7.	12/02/2006–13/02/2006
2.	24/12/2001–25/12/2001	8.	08/01/2007
3.	10/12/2003–11/12/2003	9.	08/12/2007–18/12/2007
4.	11/12/2004–14/12/2004	10.	30/11/2008
5.	24/11/2005	11.	04/01/2009–05/01/2009
6.	18/12/2005	12.	06/11/2009–07/11/2009

**Table 3:** Results of the early warning system (EWS) from the water level data determined by Kendall’s tau exceeding a threshold value of 0.9 for each CSD indicators’ variance and average spectral density at low frequencies.

Flood Events	Variance	Spectral Density
None	21/06/2000 (False alarm)	None
None	09/08/2000 (False alarm)	None
23/11/2000	18/11/2000 (EWS 5 days)	18/11/2000 (EWS 5 days)
24/12/2001–25/12/2001	04/03/2001 (EWS 9 months)	19/06/2001 (EWS 6 months)
None	None	07/07/2003 (False alarm)
10/12/2003–11/12/2003	06/10/2003 (EWS 2 months)	26/11/2003 (EWS 14 days)
None	None	21/03/2004 (False alarm)
11/12/2004–14/12/2004	14/08/2004 (EWS 4 months)	21/12/2004 (Late 10 days)
24/11/2005	20/03/2005 (EWS 8 months)	No Signal
18/12/2005	No Signal	No Signal
12/02/2006–13/02/2006	13/04/2006 (Late 2 months)	25/05/2006 (Late 3 months)
None	None	05/11/2006 (False alarm)
08/01/2007	16/08/2006 (EWS 4 months)	01/01/2007 (EWS 7 days)
None	None	28/05/2007 (False alarm)
08/12/2007–18/12/2007	No signal	13/09/2007 (EWS 3 months)
30/11/2008	24/06/2008 (EWS 5 months)	No signal
04/01/2009–05/01/2009	No signal	12/03/2009 (Late 2 months)
06/11/2009–07/11/2009	29/05/2010 (Late 6 months)	14/11/2009 (Late 8 days)
None	None	06/07/2010 (False alarm)
None	None	28/09/2010 (False alarm)

**Table 4:** Results of EWS from the water level data for different values of the quantile with their respective weights.

**Events/Quantile**	10%	11%	12%	13%	14%	15%	16%	17%	18%	19%	20%
EWS (0.5)	2	4	6	6	7	10	10	10	10	11	11
Late (0.3)	10	8	6	6	5	2	2	2	2	1	1
False (−0.2)	3	3	3	5	6	6	7	7	8	9	9
Total weight	3.4	3.8	4.2	3.8	3.8	4.4	4.2	4.2	4.0	4.0	4.0

**Table 5:** Results of EWS from the water level data with the optimum threshold at the quantile 15%.

Flood Events	Signal Detected	EWS
23/11/2000	22/11/2000	Early 1 day
None	19/01/2001	False alarm
None	16/11/2001	False alarm
24/12/2001–25/12/2001	16/12/2001	Early 8 days
None	17/12/2002	False alarm
10/12/2003–11/12/2003	30/11/2003	Early 10 days
None	30/01/2004	False alarm
11/12/2004–14/12/2004	10/12/2004	Early 1 day
24/11/2005	23/11/2005	Early 1 day
18/12/2005	15/12/2005	Early 3 days
12/02/2006–13/02/2006	12/02/2006	First day detection
08/01/2007	21/12/2006	Early 18 days
None	04/11/2007	False alarm
08/12/2007–18/12/2007	07/12/2007	Early 1 day
None	29/02/2008	False alarm
30/11/2008	29/11/2008	Early 1 day
04/01/2009–05/01/2009	02/01/2009	Early 2 day
06/11/2009–07/11/2009	06/11/2009	First day detection
